# Correction: Nanofiber applications in microbial fuel cells for enhanced energy generation: a mini review

**DOI:** 10.1039/d4ra90117g

**Published:** 2024-10-04

**Authors:** Fatma Yalcinkaya, Rafael Torres-Mendieta, Jakub Hruza, Andrea Vávrová, Lucie Svobodová, Andrea Pietrelli, Ioannis Ieropoulos

**Affiliations:** a Department of Environmental Technology, Institute for Nanomaterials, Advanced Technologies and Innovation, Technical University of Liberec Studentská 1402/2 461 17 Liberec Czech Republic fatma.yalcinkaya@tul.cz; b Department of Chemistry, Faculty of Science, Humanities and Education, Technical University of Liberec Studentská 1402/2 46117 Liberec Czech Republic; c Department of Nursing and Emergency Care, Faculty of Health Studies, Technical University of Liberec Studentská 1402/2 46117 Liberec Czech Republic; d Department of Material Science, Faculty of Mechanical Engineering, Technical University of Liberec Studentská 1402/2 46117 Liberec Czech Republic; e Université de Lyon, INSA Lyon, Université Lyon 1, Ecole Centrale de Lyon, CNRS Ampère, UMR5005 F-69621 Villeurbanne France; f Civil, Maritime and Environmental Engineering Department, University of Southampton Southampton SO16 7QF UK

## Abstract

Correction for ‘Nanofiber applications in microbial fuel cells for enhanced energy generation: a mini review’ by Fatma Yalcinkaya *et al.*, *RSC Adv.*, 2024, **14**, 9122–9136, DOI: https://doi.org/10.1039/D4RA00674G.

The authors regret that the funding information was incorrectly shown in the acknowledgements section of the original manuscript. The corrected funding acknowledgement is as shown below.

This article is based upon work from COST Action (PHOENIX, CA19123) supported by COST (European Cooperation in Science and Technology). Ioannis Ieropoulos is a Bill & Melinda Gates Foundation grantee (grant #: INV-042655).

The Royal Society of Chemistry apologises for these errors and any consequent inconvenience to authors and readers.

